# Changes in the Main Physicochemical Properties and Electrochemical Fingerprints in the Production of Sea Buckthorn Juice by Pectinase Treatment

**DOI:** 10.3390/molecules29051035

**Published:** 2024-02-28

**Authors:** Kaihua Guo

**Affiliations:** Department of Biology and Food Engineering, LyuLiang University, Lvliang 033000, China; guokhui@163.com or 20141088@llu.edu.cn

**Keywords:** sea buckthorn juice, pectinase treatment, physicochemical properties, enzymatic hydrolysis, electrochemical fingerprints

## Abstract

Enzymatic hydrolysis using pectinase is critical for producing high-yield and quality sea buckthorn juice. This study determined the optimal temperature, time, and enzyme dosage combinations to guide manufacturers. A temperature of 60 °C, hydrolysis time of 3 h, and 0.3% enzyme dosage gave 64.1% juice yield—25% higher than without enzymes. Furthermore, monitoring physicochemical properties reveals enzyme impacts on composition. Higher dosages increase soluble solids up to 15% and soluble fiber content by 35% through cell wall breakdown. However, excessive amounts over 0.3% decrease yields. Pectin concentration also declines dose-dependently, falling by 91% at 0.4%, improving juice stability but needing modulation to retain viscosity. Electrochemical fingerprinting successfully differentiates process conditions, offering a rapid quality control tool. Its potential for commercial inline use during enzymatic treatment requires exploration. Overall, connecting optimized parameters to measured effects provides actionable insights for manufacturers to boost yields, determine enzyme impacts on nutrition/functionality, and introduce novel process analytical technology. Further investigations of health properties using these conditions could expand sea buckthorn juice functionality.

## 1. Introduction

Sea buckthorn berries are known for their vibrant orange color, attributed to their high content of carotenoids such as beta-carotene and zeaxanthin [1]. In addition to carotenoids, sea buckthorn is also rich in vitamin C, fatty acids, vitamins, minerals, phenolic compounds, and dietary fiber [2]. The high levels of vitamin C, palmitoleic acid, and carotenoids like lycopene contribute to the antioxidant potential of sea buckthorn [3]. The unique composition of sea buckthorn berries has attracted significant attention from both the food industry and consumers due to its potential health-promoting properties [4,5]. Among the various forms of sea buckthorn products, juice has gained popularity as a convenient and refreshing beverage that preserves the natural goodness of the fruit [6].

The production of high-quality sea buckthorn juice involves several key steps, including fruit selection, preparation, enzymatic hydrolysis, and subsequent physicochemical analysis [7,8,9]. Enzymatic hydrolysis, specifically hydrolysis using pectinase, is a crucial process that significantly affects the yield and quality of the juice [10]. Pectinase, an enzyme derived from microorganisms such as *Aspergillus niger*, possesses the ability to break down the complex pectinaceous substances in the fruit, leading to improved juice yield and an increased extraction of bioactive compounds [11].

In recent years, researchers have focused on optimizing enzymatic hydrolysis conditions to maximize juice yield and enhance the efficiency of extracting bioactive compounds from sea buckthorn pulp [12,13,14]. Factors such as enzymatic hydrolysis temperature, hydrolysis time, and enzyme dosage have been investigated to determine their impact on the physicochemical properties of the juice [15]. Understanding these factors is crucial for optimizing the enzymatic hydrolysis process and achieving the desired product characteristics [16,17].

The physicochemical properties of sea buckthorn juice play a vital role in its sensory attributes, nutritional value, and stability [18]. Parameters such as juice yield, soluble solid content, soluble dietary fiber content, and pectin content are essential indicators of the quality and nutritional value of the juice. Juice yield reflects the degree of fruit fragmentation and utilization, while soluble solid content provides information about the sugar and nutrient content [19,20]. Soluble dietary fiber content is a key parameter for evaluating the fiber content in the juice, which is associated with various health benefits [21]. Pectin, a structural component of plant cell walls, contributes to the viscosity and texture of the juice [22,23].

Moreover, recent advances in analytical techniques have enabled the characterization of complex food systems, including the development of electrochemical fingerprinting methods [24,25]. Electrochemical fingerprints provide unique patterns of potential changes over time, reflecting the underlying chemical and physical interactions within the system [26,27,28]. These fingerprints offer a comprehensive understanding of the composition, stability, and overall quality of food and beverage products [29,30,31]. While sea buckthorn juice processing has been widely studied, the specific impacts of enzymatic treatment on physicochemical properties and the use of electrochemical fingerprints for quality monitoring represents a novel area of research. The application of electrochemical fingerprinting to track physicochemical changes during production is an emerging technique not previously explored for sea buckthorn. By linking electrochemical patterns to tangible quality indicators, it can elucidate the complex molecular transformations that determine composition and functionality [32].

Therefore, this study aimed to investigate the changes in main physicochemical properties, including juice yield, soluble solid content, soluble dietary fiber content, and pectin content, during the production of sea buckthorn juice using pectinase treatment. Additionally, the impact of enzymatic hydrolysis conditions, including temperature, time, and enzyme dosage, on these properties were evaluated through an orthogonal experimental design. Furthermore, the electrochemical fingerprints of sea buckthorn juice prepared under different enzymatic hydrolysis conditions were recorded and analyzed to gain insights into the chemical and physical transformations occurring during the production process.

## 2. Results and Discussion

**Figure 1:**
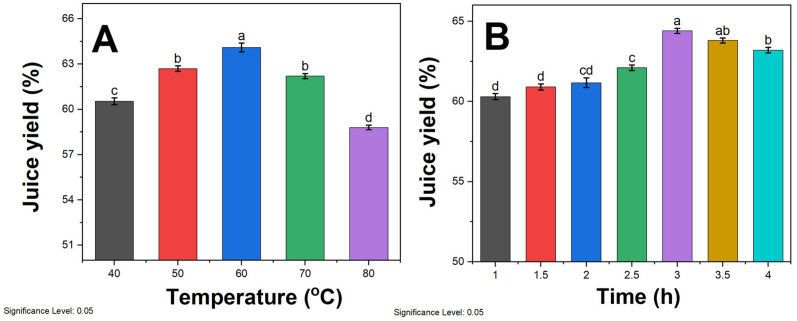
Effect of (**A**) enzymatic hydrolysis temperature at a fixed 3 h duration and (**B**) enzymatic hydrolysis time at a constant 60 °C temperature on juice yield. Enzymatic treatments were carried out by adding 0.3% pectinase solution to sea buckthorn pulp. Different lowercase letters above the bars (a, b, c, d) indicate statistically significant differences between samples. The significance level was set at 0.05.

The impact of different enzymatic hydrolysis temperatures on the juice yield of sea buckthorn pulp is depicted in Figure 1A. As the temperature rises below 60 °C, the juice yield increases with a significant difference. This phenomenon can be attributed to the enhanced permeability of cell walls and accelerated substrate movement due to higher temperatures [33]. Moreover, the activity of pectinase is correspondingly amplified, leading to the softening and degradation of pectinaceous substances in the fruit, ultimately resulting in an increased juice yield. At 60 °C, the highest juice yield was recorded to be 64.1%. Although temperatures exceeding 60 °C are not typically used in industry due to protein denaturation, we included these elevated ranges in our systematic analysis to develop a more complete understanding of temperature impacts on juice yield and pectinase mechanisms over an extensive span of potential process conditions. As the temperature continues to rise, the juice yield significantly declines. High temperatures tend to blunt or deactivate enzymes, thereby diminishing enzymatic reactions and reaction rates [34]. Additionally, prolonged exposure to high temperatures may cause the evaporation of a portion of the moisture, further reducing the juice yield. The trends in juice yield correlate well with residual pectinase activity at each respective hydrolysis temperature. After a 3 h incubation period, the 40 °C, 50 °C, 60 °C, 70 °C, and 80 °C enzyme preparations retained 95%, 92%, 87%, 63%, and 27% of their original pectolytic activity, respectively, as quantified by a viscometric assay. This indicates that thermal deactivation occurs at high temperatures. Consequently, the optimal temperature for enzymatic hydrolysis for subsequent experiments was determined to be 60 °C.

The impact of different enzymatic hydrolysis times on the juice yield of sea buckthorn pulp is illustrated in Figure 1B. When the enzymatic hydrolysis time is less than 3 h, the juice yield is directly proportional to the duration and exhibits a significant difference. However, when the enzymatic hydrolysis time exceeds 3 h, there is no significant variation in the juice yield. The process of pectinase enzymatic hydrolysis requires a sufficient amount of time. If the hydrolysis time is too short, the reaction remains incomplete, preventing the complete breakdown of cell walls and allowing them to regenerate [35], thus impeding the extraction of intracellular substances and resulting in a lower juice yield. Conversely, when the hydrolysis time is excessively long, the pectinase has already reacted completely with the substrate, and further extending the time does not significantly impact the juice yield. Moreover, prolonged enzymatic hydrolysis can lead to deteriorated taste in the mixed pulp and the loss of heat-sensitive nutrients [36]. Therefore, considering energy conservation and time cost, the optimal enzymatic hydrolysis time for subsequent experiments was determined to be 3 h.

**Figure 2:**
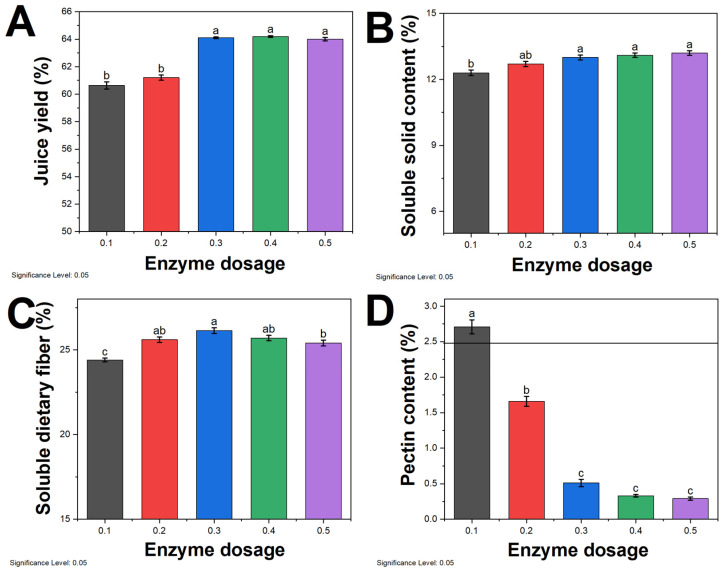
Effect of enzyme dosage on (**A**) juice yield, (**B**) soluble solid content, (**C**) soluble dietary fiber, and (**D**) pectin content. Different lowercase letters above the bars (a, b, c, d) indicate statistically significant differences between samples. The significance level was set at 0.05.

The impact of different enzyme dosage on the juice yield of sea buckthorn pulp is depicted in Figure 2A. As the enzyme dosage increases, the juice yield initially rises and then declines, reaching its peak at an enzyme dosage of 0.3%. Pectinase has the ability to break down the complex molecular chains of pulp and pectin, thereby reducing the pectin content in the juice [37]. With an increase in pectinase dosage, the pectin structure gradually breaks down, causing cell walls to rupture and releasing cellular contents, thus leading to an increase in juice yield. However, the decline in juice yield in the later stages may be attributed to several factors. An excessive pectinase dosage leads to a decrease in the effective concentration of water and reduced molecular diffusion, resulting in a lower enzymatic reaction rate [38,39]. The excessive enzymatic breakdown of pulp cells leads to the release of large amounts of sugar substances, increasing the viscosity of the juice and hindering further reactions [40]. When the pectinase concentration is too high, enzyme particles aggregate in the pulp, hindering the extraction process and making extraction difficult.

The content of soluble solids in sea buckthorn juice increases with an increase in enzyme dosage (Figure 2B). This indicates that sea buckthorn pulp obtains a greater amount of soluble solids under the action of pectinase [41]. The highest content of soluble solids is achieved when the pectinase dosage is 0.3%, and further increasing the pectinase dosage does not result in a significant increase in soluble solids. On the other hand, the content of soluble dietary fiber in the juice increases with an increase in the pectinase dosage (Figure 2C). However, after a certain point, the content of soluble dietary fiber decreases with an increase in the pectinase dosage [42]. This suggests that the dietary fiber in the juice is initially broken down into soluble dietary fiber under the action of pectinase and then further broken down into smaller non-dietary fiber components.

The pectin content in sea buckthorn juice decreases with an increase in the pectinase dosage, and the reduction is positively correlated with the pectinase dosage. When the dosage is 0.4%, the pectin content decreases by more than 90%. However, when the dosage is 0.3%, the difference in pectin content is similar to that at 0.4% pectinase dosage. Therefore, considering cost-saving factors, the optimal enzyme dosage for subsequent experiments was determined to be 0.3%.

Based on the results of single-factor experiments, three factors—temperature (A), time (B), and enzyme dosage (C)—were selected for a three-factor, three-level orthogonal experiment design. The juice yield of mixed pulp was taken as the indicator to determine the optimal enzymatic hydrolysis conditions. The experimental design and results are shown in Table 1. The range of the orthogonal experiment results reveals that the impact of the three factors on the juice yield of mixed pulp follows the order of A > B > C, indicating that temperature has the greatest influence, followed by time and enzyme dosage. The optimal combination of enzymatic hydrolysis conditions was determined to be A2B2C2, which corresponds to a temperature of 60 °C, a hydrolysis time of 3 h, and an enzyme dosage of 0.3%. To further validate the results of the orthogonal experiment, the experiment was conducted using the optimal enzymatic hydrolysis conditions, resulting in a measured juice yield of 64.1% for the mixed pulp. This represents an improvement of nearly 25% compared to the juice yield of the mixed pulp without pectinase hydrolysis.

**Figure 3:**
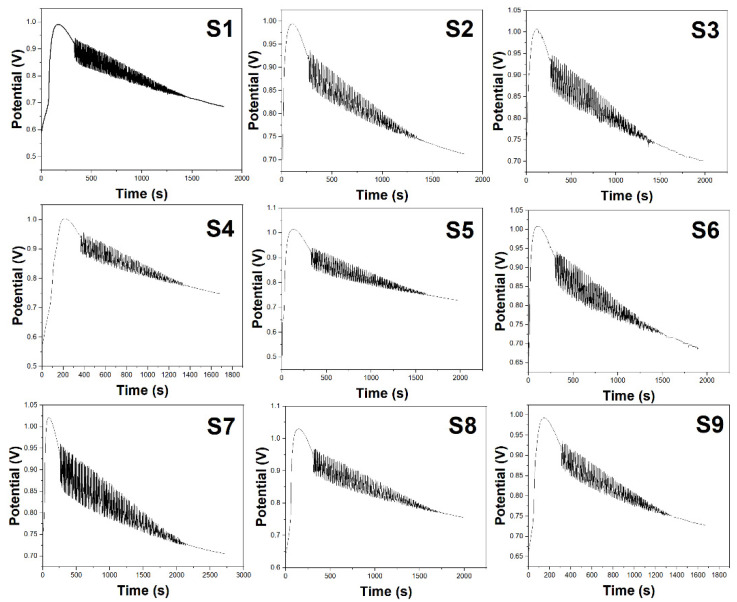
Electrochemical fingerprints of sea buckthorn prepared using the conditions listed in Table 2 (**S1**–**S9**).

Figure 3 presents the electrochemical fingerprint spectra of sea buckthorn juice prepared under the nine conditions of the orthogonal experiment mentioned above. We conducted three parallel measurements and obtained the average spectra. The characteristic parameters are listed in Table 2. Combining Figure 3 and Table 2, it is evident that the content of chemical components in sea buckthorn juice varies under different conditions, leading to distinct changes in the characteristic parameters of the oscillatory system and resulting in noticeable differences in the electrochemical fingerprint spectra. By comparing the data in Table 2, we find that S1 exhibits the longest induction time, while S2 has the shortest induction time. S7 exhibits a stoppage time exceeding 2000 s, with the highest values for oscillation lifespan, oscillation period, and maximum amplitude among the nine conditions. On the other hand, S9 has the shortest stoppage time and the lowest values for oscillation lifespan, oscillation period, and maximum amplitude. S7 has the highest values for both the initiation potential and stoppage potential, while S1 has the lowest stoppage potential.

The electrochemical fingerprint spectra provide insights into how the different enzymatic hydrolysis conditions impact the phytochemical content of the sea buckthorn juice. Comparing spectra S1–S9 reveals noticeable differences in waveform characteristics like oscillation lifespan, amplitude, and induction time. These signals are directly correlated to the levels of bioactive compounds. When considered alongside the juice yield data from the single-factor experiments (Figure 1) and orthogonal test (Table 1), some key trends emerge. For example, the higher temperature of 70 °C in S7 likely caused more rupturing of the fruit pulp cells, releasing increased quantities of intracellular metabolites. This resulted in S7 having the highest values for oscillation duration, amplitude, and juice yield among all the samples. However, as can be observed in Figure 1A, temperatures above 60 °C also led to enzyme deactivation, which decreased yields. This explains the lower juice output of S7 despite its rich phytochemical content. Meanwhile, the short hydrolysis time of just 1 h for S1 and S3 produced fewer breakdown products, reflected in their lower maximum amplitudes. Yet, S1 had the longest induction time, indicating abundant thermo-stable antioxidants like flavonoids resisting oxidation. As per Figure 2A, higher pectinase doses above 0.3% (S3) also reduced yields, possibly by increasing viscosity. Overall, the spectra parameters corroborate the patterns for factors like time, temperature, and dosage determined from juice yield analysis. S5, produced under optimal conditions, displays high amplitudes, durations, and output. Thus, electrochemical fingerprints serve as rapid indicators of preparative impacts on antioxidant quality and quantity.

## 3. Experimental Section

### 3.1. Reagents and Instruments

Sea buckthorn fruits are available on the market. Pectinase (derived from *Aspergillus niger* A3.1, 2500 U/g) was procured from Fiuka Company (Finnentrop, Germany). α-D-Galacturonic acid (98%) was purchased from Beijing Kesenlai Technology Co., Ltd., Beijing, China. Xylose (98%) was acquired from the Chinese Institute for Biological Products Inspection (Beijing, China). Folin–Ciocalteu phenol reagent was obtained from Beijing Lanboste Biotechnology Co., Ltd., Beijing, China. All other reagents used were domestically produced analytical-grade reagents.

### 3.2. Determination of Juice Yield

The juice yield is a crucial parameter to measure in the production of fruit juice beverages. It reflects the degree of fruit fragmentation and utilization. A higher juice yield indicates a greater degree of fruit fragmentation, reduced losses, and improved enzymatic activity [43]. After enzymatic hydrolysis, enzyme inactivation, and cooling to room temperature, a mixture was centrifuged at 5000 r/min for 20 min, and the supernatant was collected for calculation. Specifically, juice yield (%) was calculated gravimetrically using the following equation:

Juice yield (%) = (Total mass of juice obtained/Initial mass of fruit pulp) × 100

The total juice mass was measured after separation from the residual pomace by centrifugation (Section 3.3). The initial fruit pulp mass refers to the 100 g aliquots used for each enzymatic hydrolysis experiment. Defining juice yield in this manner allows for a systematic comparison across different process conditions to determine their impact on juice output and fruit utilization efficiency.

### 3.3. Preparation Process of Sea Buckthorn Juice

The sea buckthorn fruits used in this study were purchased fresh from a local market. A single 25 kg batch of plump, undamaged fruits was obtained to provide uniform raw material for all experiments. The preparation process of sea buckthorn juice involves several key steps. First, 2 kg of sea buckthorn fruits were selected and thoroughly washed under running water to remove dirt and impurities. Then, the fruits were crushed into a pulp using a commercial fruit pulper (Linxiao Juicer Model X20, Linxiao Machinery Co., Ltd., Taizhou, China) operating at 850 rpm rotational speed and equipped with a 0.5 mm stainless steel sieve to remove skins and seeds. The separated pulp (about 1.2 kg) was collected, and the pH was adjusted to 4.0 using a dilute sodium bicarbonate solution (Aladdin Reagent Co., Ltd., Shanghai, China). All enzymatic hydrolysis experiments were then carried out using 100 g batches of this pulp. A 10% pectinase solution was prepared by dissolving the enzyme powder in sterile distilled water at a concentration of 60,000 U/mL and activated at 37 °C for 10 min in a temperature-controlled water bath. This pectinase contains mainly pectintranseliminase, polygalacturonase, and pectinesterase and small amounts of hemicellulases and cellulases. Pectinases hydrolyze pectin, which is a component of the cell wall. They may attack methyl-esterified pectin or de-esterified pectin. The cell wall is a source of pectinase activity, also containing cellulase and hemicellulase activities. Pectinase catalyzes the random hydrolysis of a-(1-4)-Dgalactosiduronic linkages in pectin and other galacturonans. After activation, the enzyme solution was added to the pulp and thoroughly mixed, and enzymatic hydrolysis was allowed to proceed under the predetermined optimal conditions of temperature, time, and dosage. Upon completion of the allotted hydrolysis time, the enzyme was immediately deactivated by heating to 90 °C for 5 min to halt the reaction. After allowing the mixture to cool to room temperature, the liquid juice was separated from the residual pomace by centrifugation at 5000 rpm for 20 min. The supernatant sea buckthorn juice was then ready for further analysis or processing. The juice production process followed a single-factor experimental design to systematically analyze the impacts of enzymatic hydrolysis conditions on physicochemical properties. Enzyme dosage, hydrolysis temperature, and time were set as independent variables with 3 levels each based on typical industry ranges. All other parameters, such as pulp preparation and enzyme type, were kept constant. All experiments were performed in triplicate, and data are reported as mean ± standard deviation. A one-way analysis of variance (ANOVA) followed by Tukey’s multiple comparison test was conducted.

### 3.4. Determination of Soluble Solid Content, Soluble Dietary Fiber, and Pectin Content in Sea Buckthorn Juice

The soluble solid content in sea buckthorn was determined using an enzymatic and gravimetric method of AOAC [44]. The soluble dietary fiber was quantified using the phenol–sulfuric acid method with a spectrophotometer (UV-1800, Shimadzu Corp., Kyoto, Japan). The pectin content was measured using the carbazole colorimetric method with a spectrophotometer.

### 3.5. Electrochemical Fingerprints Recording

Electrochemical fingerprinting was performed using a three-electrode electrolytic cell setup connected to a potentiostat (CH Instruments, Bee Cave, TX, USA, model 660E). The working electrode was a 3 mm diameter glassy carbon disk. The reference electrode was Ag/AgCl (3 M KCl), and the counter electrode was a platinum wire. Electrochemical fingerprinting was performed using an electrolytic cell containing a mixed solution prepared by adding 3.0 M sulfuric acid (12.0 mL) (Aladdin Reagent Co., Ltd., Shanghai, China), 0.005 M cerium ammonium sulfate solution (3.0 mL) (Aladdin Reagent Co., Ltd., Shanghai, China), and 0.4 M malonic acid solution (6.0 mL) (Aladdin Reagent Co., Ltd., Shanghai, China) in a thermostatic bath set at 310 K with magnetic stirring at 600 rpm. After the electrolyte reached a steady state, sea buckthorn juice sample (0.1 mL) and 0.2 M sodium bromate solution (3.0 mL) (Aladdin Reagent Co., Ltd., Shanghai, China) were injected simultaneously to initiate the reaction. The change in potential over time was monitored using a potentiostat connected to a three-electrode system until oscillations ceased and the signal stabilized. Characteristic parameters such as induction time, oscillation lifespan, and amplitude were obtained from the dynamic multi-potential time curves. Three parallel measurements were taken for each sample, and the average fingerprint spectra were analyzed.

## 4. Conclusions

This study investigated the impacts of pectinase treatment conditions on the physicochemical properties and quality attributes of sea buckthorn juice during production. Our systematic analysis of factors like temperature, time, and enzyme dosage showed that 60 °C, 3 h, and 0.3% pectinase levels gave an optimal juice yield, as well as optimal soluble solids, dietary fiber, and pectin contents. Higher temperatures increased cell wall breakdown, improving yields initially but decreasing output and pectinase activity above 60 °C. Longer incubation times also promoted greater hydrolysis up to 3 h. However, diminished returns occurred beyond this duration. While higher enzyme doses released more soluble components through enhanced pulp disruption, excessive amounts above 0.3% lowered yields likely due to increased viscosity. Monitoring physicochemical indicators revealed enzymatic mechanisms and guided process optimization. Pectin levels declined dose-dependently with a reduction of over 90% at 0.4% dosage, improving juice stability but requiring modulation to retain viscosity. Soluble solids and dietary fiber increased with cell wall fragmentation but declined at very high doses. Introducing electrochemical fingerprinting linked measurable properties to underlying molecular changes. Unique dynamic multi-potential profiles provided rapid insights into alterations in phytochemical contents from different preparation conditions. Overall, connecting optimized parameters to observed effects enables the tailored modulation of nutrition, functionality, and bioactive components in sea buckthorn juice. The findings of this study contribute actionable guidelines for manufacturers to enhance yields, determine enzyme impacts, and introduce novel quality control technology. The further exploration of health attributes using these optimized conditions could expand sea buckthorn juice functionality.

## Figures and Tables

**Table 1:** Orthogonal test results.

Test	A (°C)	B (h)	C (%)	D (Blank)	Juice Yield (%)
S1	1 (50)	1 (1)	1 (0.2)	1	60.61
S2	1	2 (3)	2 (0.3)	2	63.70
S3	1	3 (5)	3 (0.4)	3	63.45
S4	2 (60)	1	2	3	62.33
S5	2	2	3	1	63.21
S6	2	3	1	2	63.47
S7	3 (70)	1	3	2	59.05
S8	3	2	1	3	61.50
S9	3	3	2	1	61.48
K_1_	62.55	60.57	61.80	62.11	
K_2_	63.32	63.13	62.55	62.08	
K_3_	60.70	62.50	62.21	62.41	
R	2.67	2.44	0.62	0.37	
Best level	A2	B2	C2	D3	

**Table 2:** The characteristic parameters of electrochemical fingerprints of S1–S9.

Test	t_induction_ (s)	t_stop_ (s)	t_oscillation_ (s)	τ_oscillation_ (s)	ΔE_max_ (V)	ΔE_strat_ (V)	ΔE_stop_ (V)
S1	322.15	1477.52	1170.40	9.23	0.0780	0.9211	0.7170
S2	250.42	1432.51	1182.23	10.55	0.0705	0.9380	0.7395
S3	252.51	1488.15	1233.51	9.61	0.0833	0.9421	0.7355
S4	341.50	1436.33	1095.52	9.12	0.0741	0.9285	0.7651
S5	313.75	1710.40	1420.51	10.50	0.0862	0.9257	0.7422
S6	285.70	1525.81	1240.33	9.05	0.0917	0.9442	0.7205
S7	262.65	2215.50	1950.55	13.53	0.1033	0.9551	0.7241
S8	600.95	1783.06	1481.06	12.51	0.0803	0.9242	0.7691
S9	298.17	1390.48	1091.57	9.33	0.0688	0.9251	0.7431

## Data Availability

The data presented in this study are available on request from the corresponding author. The data are not publicly available due to due to privacy.
